# New Coarse-Grained Models for Stratum Corneum Ceramides Reveal Headgroup-Dependent Structural Organization

**DOI:** 10.1021/acs.jpcb.5c05845

**Published:** 2025-11-13

**Authors:** Chloe O. Frame, Parashara Shamaprasad, Shubham Deshpande, Co D. Quach, Lingfeng Gui, Christopher R. Iacovella, Annette L. Bunge, Clare McCabe

**Affiliations:** † Department of Chemical and Biomolecular Engineering, 3120 Vanderbilt University, Nashville, Tennessee 37235, United States; ‡ School of Engineering and Physical Science, Heriot-Watt University, Edinburgh EH14 4AS, U.K.; § Department of Chemical and Biological Engineering, 3557 Colorado School of Mines, Golden, Colorado 80401, United States

## Abstract

The stratum corneum (SC), the outermost layer of human skin, owes its barrier function to highly ordered lipid lamellae composed primarily of ceramides (CERs), cholesterol (CHOL), and free fatty acids (FFAs). Molecular dynamics simulations offer the opportunity to gain valuable insights into the structural organization of lipids, complementing experimental approaches. However, simulations using atomistic models are computationally expensive when studying the large, multilamellar structures characteristic of the SC. Coarse-grained (CG) models of SC lipids provide an efficient alternative but have largely been limited to CER NS. In this work, a previously developed Multistate Iterative Boltzmann Inversion (MS-IBI) CG model for CER NS has been extended to three additional CER subclasses—CERs NP, AP, and AS—which differ from CER NS in headgroup hydroxylation. By leveraging structural similarity and transferring nonbonded interaction parameters for CER NS hydroxyl groups, we have developed new models with minimal reparameterization. The models have been validated against atomistic simulations of both pure and mixed bilayers containing CHOL and FFA. To capture the multilamellar organization, six-leaflet multilayers were self-assembled. The resulting CG systems exhibited lamellar organization, chain order, and repeat distances consistent with the available experimental data. Comparisons across CER subclasses revealed that headgroup hydroxylation influences lipid packing, chain tilt, and whether the CER tails exhibit a hairpin or extended conformation. This work demonstrates the flexibility and transferability of the MS-IBI approach and provides CG models for key CER subclasses in human SC, enabling large-scale simulations of realistic SC lipid compositions beyond the reach of atomistic models.

## Graphical abstract

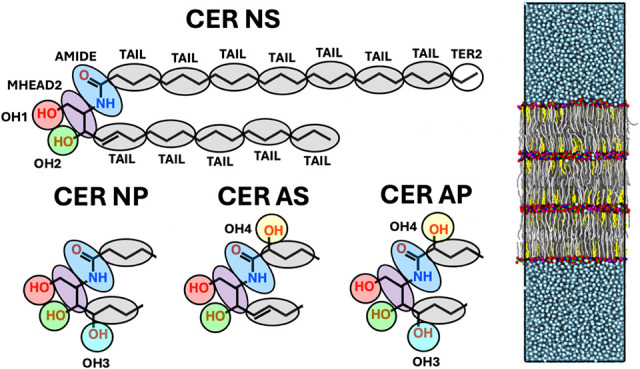

## 1 Introduction

The outermost layer of human skin, the stratum corneum (SC), plays a critical role in protecting the body from environmental and biological agents and in preventing water loss.^1^ Its effectiveness stems largely from its unique lipid composition, particularly the presence of ceramides (CERs) and the absence of phospholipids, which distinguish it from other biological membranes. In the SC, CER mixtures with cholesterol (CHOL) and free fatty acids (FFAs) form two highly ordered lamellar structures that are essential for SC barrier function—namely, the short and long periodicity phases (SPP and LPP), with repeat distances of approximately 6 and 13 nm, respectively.^1–5^ Both human SC and synthetic lipid models designed to replicate SC composition and structures include an array of CER subclasses that vary in headgroup chemistry as well as tail structure and length.

Molecular dynamics (MD) simulations can be effective tools for investigating the structural and dynamic behaviors of lipid systems at the atomic level. MD simulations of SC lipid structures require selecting an appropriate model or force field that describes the molecules of interest and defines the parameters governing the bonded and nonbonded molecular interactions. Almost all atomistic simulations of SC lipids have utilized force fields from either the CHARMM or GROMOS frameworks, often with minor parameter adjustments to better match ab initio calculations or experimental data of specific lipid structures and dynamics.^6^ CHARMM^7,8^ employs an all-atom (AA) approach that explicitly represents every atom, whereas GROMOS^9^ uses a united atom model that groups CH_*n*_ units into a single interaction site.

Many of the atomistic simulations of SC lipid systems reported in the literature have examined the SPP in preassembled structures of either bilayers (two leaflets) or multilayers (stacked leaflets) that contain CER NS (a nonhydroxy acyl tail bonded to a sphingosine base, see Figure 1), even though it is not the most abundant CER in human SC.^1^ However, more recent experiments^10–15^ and simulations^6^ have examined systems containing CERs other than CER NS, including CER NP (a nonhydroxy acyl chain bonded to a phytosphingosine base), CER AS (an α-hydroxy acyl chain bonded to a sphingosine base), and CER AP (an α-hydroxy acyl chain bonded to a phytosphingosine base). In an extensive atomistic simulation study of different CER subclasses, Badhe et al.^6,16^ used the GROMOS-Notman force field to simulate preassembled bilayers of pure CERs (including NP, AS, AP, and three others), each with 24-carbon acyl and 18-carbon sphingoid base chains. The lipids of the SC exist in highly ordered gel phases, exhibiting slow dynamics.^6^ As a result, preassembled simulations can be biased toward the initial lipid configuration used in the simulation.^6,17^ Furthermore, recent experiments show that in ternary mixtures of CERs, CHOL, and FFA, which are more representative of SC lipid composition, most CERs adopt an extended conformation (with their two tails pointing into adjacent leaflets) rather than the hairpin conformation (with their two tails pointing into the same leaflet).^1,18–21^ In bilayer simulations with water on both sides, the CERs are restricted to the hairpin conformation.

**Figure 1:**
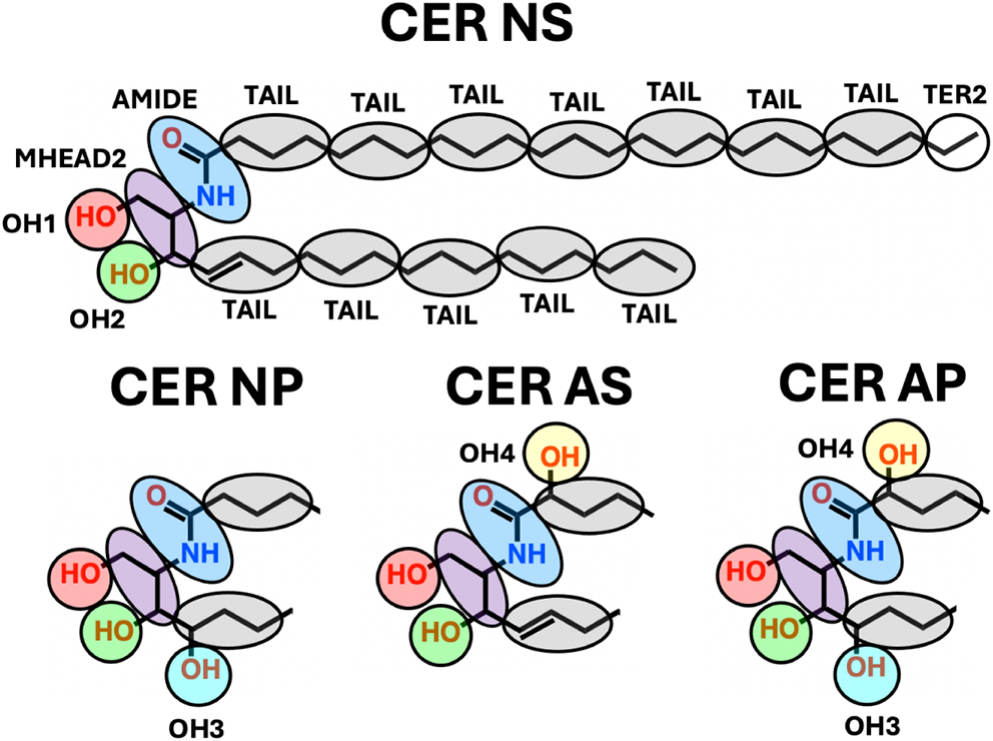
Molecular structures and CG mapping schemes for CER NS C24 and for the headgroups of CERs NP, AS, and AP. The CG mapping of CER NS identifies the CG beads (MHEAD2, AMIDE, OH1, OH2, TAIL, and TER2) used in all four CER models. The terminal bead on the sphingosine or acyl chains of CERs (and FFA; see Figure 2) is either a TAIL bead (containing two methylenes and a terminal methyl) or a TER2 bead (containing one methylene and a terminal methyl). Chain length variations are represented by differing numbers of TAIL beads and either a TAIL or a TER2 terminal bead.

Simulating multilayer systems where CERs can adopt an extended conformation while also ensuring that the results are not biased by the initial lipid configurations requires simulations of much larger system sizes and over longer time scales than is currently feasible with atomistic simulations. Coarse-grained (CG) models, in which 2–5 atoms and the corresponding hydrogen atoms are grouped together into a single interaction site (a CG bead), can be used to simulate large system sizes for long times due to the reduced number of interactions that need to be computed at each time step and the softening of those interactions relative to the atomistic system. However, developing accurate CG models for each of the important CER subclasses in human SC requires careful consideration of the mapping of the molecules to the CG level and the interactions between the CG beads in the model.

MARTINI-based CG force fields have been widely used to study lipids^6^ including CERs NS,^22–24^ NP,^25^ and AP.^26^ The MARTINI model offers a simplified approach for parametrizing lipid molecules by providing generalized interaction parameters that can be used to build the lipid molecule of interest. However, because the parameters are not tailored to individual molecules or molecular classes, they can fail to capture critical SC lipid structural behavior.^6^ Furthermore, since the MARTINI model was optimized based on free energy data, MARTINI simulations generally perform well in estimating thermodynamic properties, such as partitioning and interfacial tension, but can be less accurate in predicting structural properties, such as radial distribution functions (RDFs), area per lipid (APL), lipid tilt angle, and density profiles. This is potentially relevant when studying the lipids of the SC, since it is the structural properties of the lipid lamellae and the characteristic orthorhombic and hexagonal packing of the lipids that impart the SC barrier function.^1^ For instance, MARTINI simulations of bilayers of pure CER NS C24 (a 24-carbon acyl chain bonded to an 18-carbon sphingoid base) overestimate the APL (0.46 nm^2^)^23^ compared to experiments (0.38 to 0.42 nm^2^).^22,23,27^

Taking a different approach, Moore et al.^28^ used the structure-based Multistate Iterative Boltzmann Inversion (MS-IBI) method to develop CG models that are structurally accurate and state transferable. This is accomplished by optimizing nonbonded pair potentials between CG beads to reproduce RDF targets from AA simulations at multiple state points simultaneously. CG models for CER NS,^29^ CHOL,^30,31^ FFA,^29,32–34^ and water^35–37^ have been developed using the MS-IBI approach. The models minimize the number of distinct CG beads required by transferring parameters across chemically similar but distinct molecules. For example, the same CG *TAIL* beads, each representing three CH_2_ groups, are used in the long alkyl chains of both CERs and FFAs. Additionally, unlike MARTINI, chemically identical groups may be assigned different nonbonded interaction parameters when necessary to improve predictions of key structural properties, such as capturing directional headgroup interactions between lipids due to hydrogen bonding. For instance, explicitly representing the two hydroxyl groups in CER NS as distinct OH1 and OH2 beads with different nonbonded interaction parameters was essential for obtaining experimentally consistent APL values (0.4199 ± 0.0009 nm^2^).^29^

In this work, the previously parametrized CG model for CER NS was used to construct CG models of other CER subclasses without extensive reparameterization. This strategy streamlines model development for new molecules while maintaining accuracy. We describe here how the MS-IBI CG-derived parameters for CER NS are extended to three of the most abundant CERs in human SC: CERs NP, AP, and AS. The headgroups of these CERs are similar to those of CER NS, with differences in the number and placement of hydroxyl groups. We demonstrate that the nonbonded potentials for one of the OH beads in the CER NS model can be applied to the two new OH beads representing additional hydroxyl groups in CERs NP, AP, and AS. The resulting CG models are tested and validated through comparisons with atomistic simulations. Self-assembly simulations of both bilayers and multilayers were conducted for each CER subclass as well as for each CER subclass in a ternary lipid mixture with CHOL and FFA, to evaluate model performance and examine how the different CER headgroups influence lipid organization and structure.

## 2 Methods

Both AA and CG simulations were conducted for CERs in pure form and in ternary mixtures with CHOL and FFA in a molar ratio of 1:0.5:1. The CHOL composition in the ternary mixtures was half of that used in many simulated and experimental systems, as experimental studies show phase separation of CHOL at equimolar CER:CHOL:FFA ratios. This behavior disappears when the CHOL amount is reduced to the lower molar ratio used in our simulations.^21,38–40^ These findings suggest that the lamellar phase composition in these experiments is likely closer to the CER:CHOL:FFA molar ratio of 1:0.5:1 and thus the composition studied herein is more representative of the experimental systems. In all simulations, the acyl and sphingoid base chains of CER molecules contained 24 and 18 carbons, respectively. FFA chains were 24 carbons in length, saturated, and fully protonated.

### 2.1 Coarse-Grained Mapping and Bead Interaction Parameters

Schemes for mapping the atoms in the SC lipids to CG beads are shown in Figure 1 for CER NS C24 and in Figure 2 for CHOL and FFA C24. CG water beads contain on average four dynamically mapped water molecules, consistent with previous studies.^29,30,35^ Atoms in the CER NS headgroup are mapped onto four beads: AMIDE, MHEAD2, OH1, and OH2. The two hydroxyl groups are represented as distinct OH1 and OH2 beads with separate interaction parameters to account for their directional hydrogen-bonding behaviors. The sphingosine and acyl chains of CER NS, as well as the FFA tail, are represented by TAIL and TER2 beads, the terminal group used being dictated by the chain length (i.e., with a 3:1 mapping, an 18-carbon sphingoid base chain terminates in a TAIL bead, whereas a 24-carbon acyl chain terminates in a TER2 (2:1 mapping) bead). Following the mapping of Moore et al.,^29^ TAIL beads for alkyl carbons with and without double bonds were treated equivalently in the CG model.

**Figure 2:**
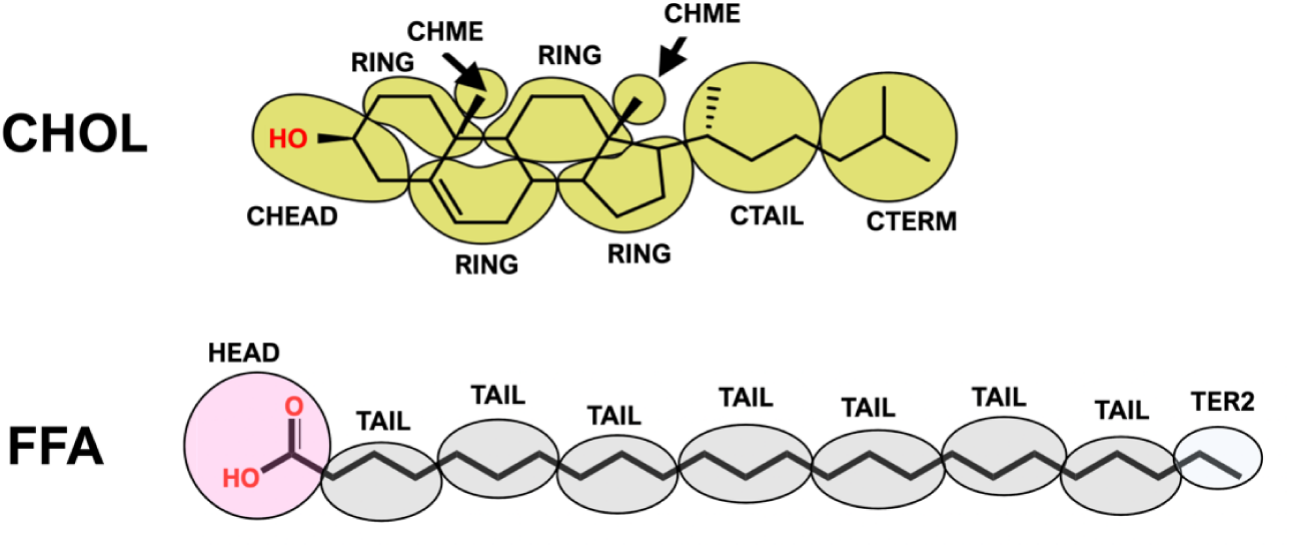
Molecular structures and CG mapping schemes for CHOL^30,31^ and FFA C24 ^29^.

Figure 1 also presents mapping schemes for the headgroups of the new CG models for CERs NP, AS, and AP, which are identical to those of CER NS except for the additional hydroxyl groups as shown. These models utilize the bonded and nonbonded interaction parameters derived for the CG beads in CER NS (i.e., the MHEAD2, AMIDE, OH1, OH2, TAIL, and TER2 beads are transferable to the new models) combined with two new beads representing separately the additional hydroxyl groups on the phytosphingosine base (OH3) and the α-hydroxy acyl chain (OH4). The positions of the additional hydroxyl groups on these headgroups are known to affect CER packing, most probably through differences in hydrogen bonding.^1^ Therefore, as with the OH1 and OH2 beads in CER NS, the new OH beads are treated as separate entities, allowing for distinct bonded and nonbonded interaction parameters. We assume that the interaction parameters for OH3 and OH4, like those for OH1 and OH2, are identical across all CER subclasses (i.e., the parameters are transferable).

Bonded interactions are characterized by the equilibrium bond length (*r*_0_) and the harmonic force constant (*k*_*r*_) for bonds between adjacent CG beads, and by the equilibrium angle (θ_0_) and the harmonic force constant (*k*_0_) for the angle between three connected CG beads. Values for these parameters are derived by Boltzmann inversion of the Gaussian distribution (*y*) of bond lengths (*r*) and angles (θ) obtained from AA simulations mapped to the CG beads, as previously described:^29,30^

$$y=\exp\left[-\frac{k}{2k_{B}T}{\left(x-x_{0}\right)}^{2}\right]$$

where *x* is either *r* or θ, *k* is either *k*_*r*_ or *k*_0_, *T* is the temperature, and *k*_B_ is the Boltzmann constant. Bond length and angle parameters were determined for OH3 from simulations of CER NP and for OH4 from simulations of CER AS. The final values were selected based on how well they reproduced the bond length and angle distributions from AA simulations of pure CER NP and AS bilayers mapped to the CG level. Due to transferability, these parameters are also used for the OH3 and OH4 beads in CER AP.^29^

The nonbonded interaction parameters for the CG beads in CER NS, FFA, and CHOL were derived using the MS-IBI method, combined with simulations of wetting at the lipid–water interface to achieve the appropriate hydrophilic–lipophilic balance.^29,34^ As a first step toward parametrizing the nonbonded interactions, the OH1 and OH2 beads were tested for their ability to represent the OH3 and OH4 beads, which would minimize the addition of new bead types and maximize the transferable nature of the CG model. Every combination of OH1 or OH2 parameters was considered for OH3 in CERs NP and AP and OH4 in CERs AS and AP. The suitability of each option was assessed based on whether the resulting CG models could self-assemble into well-organized bilayers and multilayers, both in pure form and in ternary mixtures with CHOL and FFA, and whether the structural properties of the CG bilayers reasonably matched those from preassembled AA simulations of the same system. All nonbonded and bonded interaction parameters for the CG models presented in Figures 1 and 2, including those developed in this work, are provided in a GitHub repository.^41^

### 2.2 All-Atom Simulations

AA simulations were conducted to provide targets for the derivation of the CG bonded interaction parameters for the OH3 and OH4 beads as well as to obtain structural properties against which to validate the CG models. Simulations were performed on preassembled bilayers of pure CERs and ternary lipid mixtures of each CER with CHOL and FFA C24 (1:0.5:1 molar ratio), ensuring that the configurations are equilibrated away from the initial configuration as described below.

The simulations employed the CHARMM36 force field, incorporating additional parameters from Cournia et al.^42^ for CHOL, along with the TIP3P water model.^41,43^ CER headgroup parameters were taken from Guo et al.^27,41^ for CERs NS and NP and used to initialize models of CERs AP and AS, with charge distributions adjusted to account for additional hydroxyl groups. Topology and force field files for CERs NS, NP, AS, and AP are provided in a GitHub repository.^41^ Bilayers were preassembled using the MoSDeF software suite^44–51^ with pure CER systems containing 128 lipids and CER:CHOL:FFA mixtures (at a 1:0.5:1 molar ratio) containing 200 lipids. Each bilayer was hydrated by two equal layers of water with a total of 40 water molecules per lipid.

The AA simulations were performed using GROMACS 2020.6 ^52^ with a 1 fs time step and periodic boundary conditions in all three directions. van der Waals and short-range electrostatic interactions were truncated at 1.2 nm, while long-range electrostatic interactions were treated with the particle-mesh Ewald (PME) method using a 1.2 nm cutoff. Simulations began with energy minimization using the steepest descent algorithm for at most 200,000 steps to resolve high-energy lipid overlaps. The bilayers were then equilibrated in the canonical (NVT) ensemble for 500 ps at 305 K using the Nosé–Hoover thermostat,^53^ followed by equilibration in the isothermal–isobaric (NPT) ensemble at 305 K and 1 bar for 10 ns using the Parrinello–Rahman barostat^54^ with semi-isotropic pressure coupling (applied in the *x*–*y* plane) to maintain bilayer shape. To allow the preassembled systems to escape their initial configurations and potential metastable states, the random walk molecular dynamics (RWMD) algorithm^55^ was applied for 100 ns, cycling temperatures between 305 and 350 K. Finally, a 20 ns production run was performed in the NPT ensemble at 305 K, with trajectories recorded every 10 ps, yielding 2000 frames for analysis.

### 2.3 Coarse-Grained Simulations

CG simulations were conducted on both self-assembled bilayers and six-leaflet multilayers of either pure CERs or mixtures of each CER subclass with CHOL and FFA. Lipids were randomly packed between equal amounts of water using mBuild,^51^ with water added at a density of 1 g/cm^3^ for a total of 10 CG water beads per lipid (equivalent to 40 water molecules per lipid as in the atomistic simulations). This configuration promotes the alignment of lipid leaflets parallel to the *x*–*y* plane of the simulation box. The initial simulation box dimensions were determined based on a lipid density of 0.8 g/cm^3^ and a cross-sectional area suitable for forming the desired number of leaflets (either two or six). Bilayer simulations included 500 lipids for pure CERs (NP, AS, and AP) and 1800 lipids for their mixtures with CHOL and FFA (1:0.5:1 molar ratio). Six-leaflet multilayer simulations contained 1800 lipids.

Simulations were performed using HOOMD-Blue^56,57^ version 2.9.7 with a 10 fs time step and periodic boundary conditions in all three dimensions. The simulations began with a 10 ps run in the constant energy, constant volume (NVE) ensemble at 105 K using the Velocity-Verlet integration algorithm, with a 0.1 Å displacement limit of each bead at every time step to eliminate high-energy molecular overlaps. This was followed by a second 10 ps NVE run at 305 K. Next, the system density was equilibrated in the NPT ensemble for 10 ns at 305 K and 1 bar using the Martyna–Tobias–Klein (MTK) barostat–thermostat^58^ for integration. After equilibration, a shape annealing procedure was employed to accelerate the self-assembly and enable the system to escape metastable states. Originally proposed by Moore et al.,^29,34^ this procedure involves expansion and compression of the simulation box area relative to its estimated value (*A*_est_), while maintaining constant volume. *A*_est_ is calculated as

$$A_{est}=APL_{est}N_{lipids}/N_{leaflets}$$

where *N*_lipids_ is the number of lipids, APL_est_ is an estimate of the area per lipid, and *N*_leaflets_ is the number of leaflets (i.e., two or six). In this procedure, the simulation box volume remained constant in the NVT ensemble, using the Nosé–Hoover thermostat to control the temperature. The area was expanded to 2.5 times *A*_est_ over 150 ns at 500 K, then compressed back to *A*_est_ with a linear temperature ramp from 500 to 305 K over 150 ns. The value chosen for APL_est_ was found to not be critical as long as it was reasonable. Here, APL_est_ was set to 0.34 nm^2^, except for multilayers of pure CERs AS and AP, where APL_est_was increased to 0.44 nm^2^ to avoid occasional eight-leaflet formation.

Following shape annealing, the system was returned to the NPT ensemble. Using a semi-isotropic MTK barostat–thermostat^58^ with a constant pressure of 1 bar and pressure coupling applied in the *x*–*y* plane, the temperature was increased from 305 to 400 K over 100 ns at a constant rate, then reduced to 305 K over 50 ns at a constant rate. This heating–cooling step minimized defects formed during shape annealing and further relaxed the lipids. Finally, the simulation was run for 150 ns under physiological conditions at 305 K and 1 bar. The stability of the equilibrated CG structure was confirmed by a constant simulation box width. The last 100 ns of the simulation was used as the production run, with a trajectory recorded every 0.5 ns, yielding 200 frames for analysis. In total, the self-assembly procedure took approximately 1–2 μs.

### 2.4 Analysis

Several structural properties were calculated from the CG and AA simulations. The APL was determined by dividing the cross-sectional area of the simulation box by the number of lipids in each leaflet. Lipid packing was assessed using the normalized lipid area (NLA) proposed by Shamaprasad et al.,^30^ which accounts for lipid size differences by scaling the APL based on the effective number of hydrocarbon tails per lipid, estimated from the ratio of the cross-sectional areas of each lipid relative to that of a saturated hydrocarbon chain: 1 for FFAs, 2 for CERs, and 1.9 for CHOL. Bilayer thickness and the leaflet-pair thickness in six-leaflet multilayers were measured as the peak-to-peak distance in the mass density profile. The lipid tilt angle is defined as the angle between the bilayer normal (*z*-axis) and the long axis of the lipid tails (i.e., the axis with the least resistance to rotation).^59^ This axis was determined from the eigenvector corresponding to the minimum eigenvalue of the inertia tensor.^60^ The nematic order parameter (*S*_2_) was obtained from the largest eigenvalue of the nematic tensor, following the method described in Wilson.^61^ Interdigitation, λ, was determined from the mass density profiles of the central leaflet pair of the six-leaflet multilayer membranes, with “top” and “bottom” referring to lipids with headgroups located above and below the simulation box midpoint, respectively and is calculated using the following equation from Das et al.^62^

$$\lambda =4\int_{z_{min}}^{z_{max}}\frac{\rho_{top}\left(z\right)\rho_{bot}\left(z\right)}{{\left(\rho_{top}\left(z\right)+\rho_{bot}\left(z\right)\right)}^{2}}dz$$

Here *ρ*_top_(*z*) and *ρ*_bot_(*z*) represent the mass density distributions of the top and bottom lipids as functions of the *z*-coordinate. The limits of integration, *z*_min_ and *z*_max_, correspond to the minimum and maximum *z* values of the simulation box. CER molecules were considered to be in an extended conformation when the angle between the directional vectors associated with the centers of mass of the acyl and sphingosine tails was greater than 90°.

Simulation results were averaged over all leaflets in each frame and then across all frames from the simulation. All results, except the tilt angle, are reported as the average and standard deviation calculated from replicate simulations with different randomized initial configurations. For the tilt angle, which is calculated for each lipid, the standard deviation is reported as the square root of the variance, where the variance is calculated from the tilt angle of the molecules in each leaflet, of each frame, of each replicated simulation, and then pooled across all leaflets, frames, and replicates. Details are provided in the Supporting Information. Bilayer simulations were repeated four times from different randomized initial configurations; six-leaflet multilayer simulations were repeated four times for pure CERs and three times for CER mixtures with CHOL and FFA.

## 3 Results and Discussion

Structural properties from AA bilayer simulations are first compared to prior AA simulation and experimental data to ensure their suitability as validation targets for CG model development. Next, the bonded and nonbonded interaction parameters needed to extend the CG CER NS model to CERs NP, AS, and AP are defined. The new CG models are then used to examine how the CER headgroup affects lamellar structure and organization, both in pure CER multilayers and in ternary mixtures with CHOL and FFA.

### 3.1 Atomistic Bilayer Structures Compared with Prior Simulations and Experiments

Structural properties from AA simulations of preassembled bilayers of pure CERs NP, AS, and AP, as well as their mixtures with CHOL and FFA, are presented in Tables 1 and 2, respectively, and compared with previously published results for CER NS from similar simulations. Among the pure CER bilayers, CER AP has the largest APL (0.427 ± 0.012 nm^2^), while CERs NP and AS have similar values (0.405 ± 0.005 nm^2^ and 0.402 ± 0.004 nm^2^, respectively). The APLs of CERs NP and AS also closely match previously reported values for CER NS (0.399 ± 0.002 nm^2^ at 305 K).^29^ As expected, APL values are lower in CER mixtures (∼0.31 nm^2^ vs ∼0.41 nm^2^) due to the presence of FFA, which has a single acyl tail and a correspondingly smaller APL. However, the NLA values, which account for the size differences of CER, CHOL, and FFA, are nearly identical to those of the pure CERs.

**Table 1:** Structural Properties for Pure CER Bilayers from Preassembled AA and Self-Assembled CG Simulations^a^

		OH3/OH4 Bead Types						
CER	Simulation Type	OH3	OH4	APL (nm^2^)	NLA (nm^2^)	Tilt Angle (°)	Bilayer Thickness (nm)	S_2_
NP	AA			0.405 ± 0.005	0.203 ± 0.002	11.6 ± 2.8	5.36 ± 0.13	0.98 ± 0.01
	CG	OH1	–	0.426 ± 0.003	0.213 ± 0.001	8.0 ± 2.8	5.16 ± 0.03	0.97 ± 0.01
		OH2	–	0.426 ± 0.002	0.213 ± 0.001	9.0 ± 2.5	5.16 ± 0.02	0.97 ± 0.01
AS	AA			0.402 ± 0.004	0.201 ± 0.002	13.4 ± 1.0	5.34 ± 0.07	0.97 ± 0.01
	CG	–	OH1	0.428 ± 0.002	0.214 ± 0.001	9.6 ± 2.5	5.20 ± 0.03	0.98 ± 0.01
		–	OH2	0.428 ± 0.001	0.214 ± 0.001	10.4 ± 3.6	4.76 ± 0.78	0.95 ± 0.07
AP	AA			0.427 ± 0.012	0.210 ± 0.010	15.5 ± 4.9	5.01 ± 0.08	0.97 ± 0.02
	CG	OH1	OH1	0.443 ± 0.006	0.222 ± 0.003	15.9 ± 5.5	5.03 ± 0.06	0.91 ± 0.08
		OH1	OH2	0.443 ± 0.001	0.222 ± 0.000	18.0 ± 4.2	5.05 ± 0.02	0.90 ± 0.08
		OH2	OH1	0.436 ± 0.004	0.218 ± 0.002	12.6 ± 3.6	5.09 ± 0.03	0.95 ± 0.03
		OH2	OH2	0.437 ± 0.003	0.218 ± 0.001	14.9 ± 4.9	5.13 ± 0.02	0.90 ± 0.09
NS^b^	AA	–	–	0.399 ± 0.002	0.200 ± 0.001	22 ± 4^c^	5.62 ± 0.04^d^	0.98
	CG	–	–	0.420 ± 0.002	0.210 ± 0.001	7 ± 1^c^	5.66 ± 0.01^d^	0.98

a Results are presented for the CG models using different combinations of the OH1 and OH2 beads’ nonbonded interaction parameters for the OH3 and OH4 beads.

b From Moore et al.^29^

c The tilt angle was calculated as the angle between the tail director vector and the bilayer normal, which differs from the method used in this study; this difference in methodology should be considered when comparing results.

d Bilayer thickness was calculated as the distance between the positions where the water density falls to 1/e of the bulk value along the normal at the lipid–water interfaces. This method yields systematically larger thickness than the peak-to-peak distance in the mass density profile used in this study.^6^

**Table 2:** Structural Properties for Bilayers of CER:CHOL:FFA Mixtures with 1:0.5:1 Molar Ratio from Preassembled AA and Self-Assembled CG Simulations^a^

		OH3/OH4 Bead Types						
CER	Simulation Type	OH3	OH4	APL (nm^2^)	NLA (nm^2^)	Tilt Angle (°)	Bilayer Thickness (nm)	S_2_
NP	AA			0.304 ± 0.006	0.192 ± 0.004	10.3 ± 1.2	5.37 ± 0.11	0.94 ± 0.00
	CG	OH1	–	0.331 ± 0.001	0.210 ± 0.001	7.8 ± 0.1	5.05 ± 0.01	0.96 ± 0.00
		OH2	–	0.332 ± 0.000	0.210 ± 0.000	7.9 ± 0.2	5.04 ± 0.00	0.96 ± 0.00
AS	AA			0.306 ± 0.003	0.193 ± 0.002	8.3 ± 0.6	5.34 ± 0.08	0.97 ± 0.00
	CG	–	OH1	0.323 ± 0.001	0.208 ± 0.001	7.6 ± 0.3	5.10 ± 0.01	0.97 ± 0.00
		–	OH2	0.330 ± 0.001	0.209 ± 0.000	7.9 ± 0.2	5.10 ± 0.01	0.96 ± 0.00
AP	AA			0.308 ± 0.003	0.195 ± 0.002	13.3 ± 1.3	5.30 ± 0.05	0.89 ± 0.01
	CG	OH1	OH1	0.332 ± 0.000	0.210 ± 0.000	8.5 ± 0.2	5.03 ± 0.00	0.95 ± 0.00
		OH1	OH2	0.334 ± 0.000	0.211 ± 0.000	8.7 ± 0.2	5.03 ± 0.00	0.95 ± 0.00
		OH2	OH1	0.333 ± 0.000	0.211 ± 0.000	8.4 ± 0.3	5.02 ± 0.00	0.96 ± 0.00
		OH2	OH2	0.336 ± 0.000	0.212 ± 0.000	9.0 ± 0.1	5.02 ± 0.01	0.95 ± 0.00
NS^b^	AA	–	–	0.304 ± 0.001	0.192 ± 0.000	8 ± 4	5.27 ± 0.02	0.98 ± 0.01
	CG	–	–	0.333 ± 0.002	0.211 ± 0.001	8 ± 5	5.08 ± 0.002	0.96 ± 0.02

a Results are presented for the CG models using different combinations of the OH1 and OH2 beads’ nonbonded interaction parameters for the OH3 and OH4 Beads.

b From Shamaprasad et al.^30^

The APL values from our simulations are generally in good agreement with previously published CHARMM-based results. Guo et al.^27^ reported similar APL values for pure CERs NP and NS (0.421 ± 0.002 nm^2^ and 0.424 ± 0.002 nm^2^, respectively, at 305 K with a C16 acyl chain), although we note that their values are slightly larger, perhaps due to the shorter acyl chain length. Likewise, Wang and Klauda^63^ reported a larger APL for CER AP compared to CER NS (0.464 ± 0.001 nm^2^ vs 0.428 ± 0.002 nm^2^ at 305 K), consistent with our findings. However, their APL values are also slightly larger than those reported herein, likely due to differences in the headgroup parameters used in the simulations.^41,64^

Using the GROMOS-Berger (united atom) force field, Badhe et al.^16^ calculated lower values and a different ranking for the APLs of CERs AS, AP, and NP (0.395 nm^2^, 0.385 nm^2^ and 0.378 nm^2^, respectively at 310 K) compared with our results. The lower APL values are consistent with observations from other studies that GROMOS-based simulations produce smaller APLs than CHARMM-based ones, although some cases show comparable results. For example, Guo et al.^27^ found that the CHARMM-based force field predicted a larger APL for CER NS C16 than GROMOS (0.424 ± 0.002 vs 0.398 ± 0.002), with the CHARMM result showing better agreement with experimental monolayers. Similarly, Mistry and Notman^65^ reported smaller APL values for equimolar mixtures of CER NS with CHOL and FFA using GROMOS (0.307 ± 0.0003 nm^2^) compared to using CHARMM (0.325 ± 0.002). However, in one case, Gupta and Rai^66^ reported an APL of 0.393 nm^2^ from their GROMOS simulation of CER NS at 300 K, which is similar to the CHARMM-based value of (0.399 ± 0.002 nm^2^ at 305 K) reported by Moore et al.^29^

Pure CERs NP and AS showed similar bilayer thicknesses (5.36 ± 0.13 and 5.34 ± 0.07 nm, respectively) that were larger than that of CER AP (5.01 ± 0.08 nm). In contrast, Badhe et al.,^16^ using GROMOS, reported a thickness for CER AS (4.98 nm) that matched CER AP but was smaller than that of CER NP (5.09 nm). Although different calculation methods were used to calculate the bilayer thickness in the two studies (distance between the headgroup peaks in the mass density versus electron density profiles), the two methods should yield consistent comparisons among the CERs. Therefore, the observed discrepancies are most likely due to differences in the force fields used.

Table 2 summarizes the structural properties for bilayers of CERs NP, AS, and AP mixed with CHOL and FFA C24 in a 1:0.5:1 molar ratio. The results indicate that once CHOL and FFA are introduced, the bilayer structural properties become independent of the CER subclass. This is consistent with experimental and simulation findings from Nădăban et al., which showed that varying the relative amounts of CER NS C24 to CER NP C24 from 1:2 to 2:1 in CER:CHOL:FFA C24 mixtures (1:0.5:1 molar ratio) had minimal impact on structural organization.^38^ Similarly, Kováčik et al.^15^ observed little variation in experimental repeat distances for CERs NS, AS, and AP (all C24) in equimolar mixtures with CHOL and a blend of five FFAs (C16 to C24, average chain length ∼23 carbons), reporting distances of 5.33, 5.27, and 5.34 nm, respectively. All of the systems studied by Kováčik et al.^15^ contained phase-separated crystalline CHOL, thus reducing the CHOL content in the lipid lamellae to below equimolar levels. The bilayer thicknesses obtained herein for CER AS and AP mixtures with CHOL and FFA C24 at a 1:0.5:1 molar ratio (5.34 ± 0.08 nm and 5.30 ± 0.05 nm, respectively) are in close agreement with those reported by Kováčik et al.^15^

### 3.2 Coarse-Grained Interaction Parameters for the New OH Beads

#### 3.2.1 Bonded Interaction Parameters

Table 3 lists the bonded interaction parameters for the OH3 and OH4 CG beads. Table S1 in the Supporting Information provides parameters for all CG beads in the CER molecules. Figure 3 compares distributions of bond angles and bond lengths from CG simulations to those from AA simulations mapped to the CG representation for bilayers of pure CERs AP (Figure 3A,B,E,F), NP (Figure 3C,G), and AS (Figure 3D,H). Because of transferability, OH3 parameters developed using CER NP, and OH4 parameters developed using CER AS, are used in CER AP.

**Table 3:** Bonded Interaction Parameters for the OH3 and OH4 Beads in the CER CG Models: *r*_0_ Is the Length and *k*_*r*_ Is the Harmonic Force Constant for the Bonds between Adjacent CG Beads; *θ*_0_ Is the Angle and *k*_0_ Is the Harmonic Force Constant for the Bond Angle between Three Connected Beads

Bond	*r*_0_ [Å]	*k*_*r*_ [kcal/(mol Å^2^)]
TAIL–OH3	2.40	93.0
TAIL–OH4	2.50	542.0

**Angle**	θ_**0**_ **[degrees]**	***k***_**0**_ **[kcal/(mol-degrees** ^**2**^ **) × 10** ^**3**^]
TAIL–TAIL–OH3	143.8	16.5
TAIL–TAIL–OH4	141.0	4.88
AMIDE–TAIL–OH4	71.0	149
MHEAD2–TAIL–OH3-a^a^	52.2	70.4
MHEAD2–TAIL–OH3-b	77.3	97.5

a The MHEAD2–TAIL–OH3 angle is characterized by two distributions, labeled as a and b.

**Figure 3:**
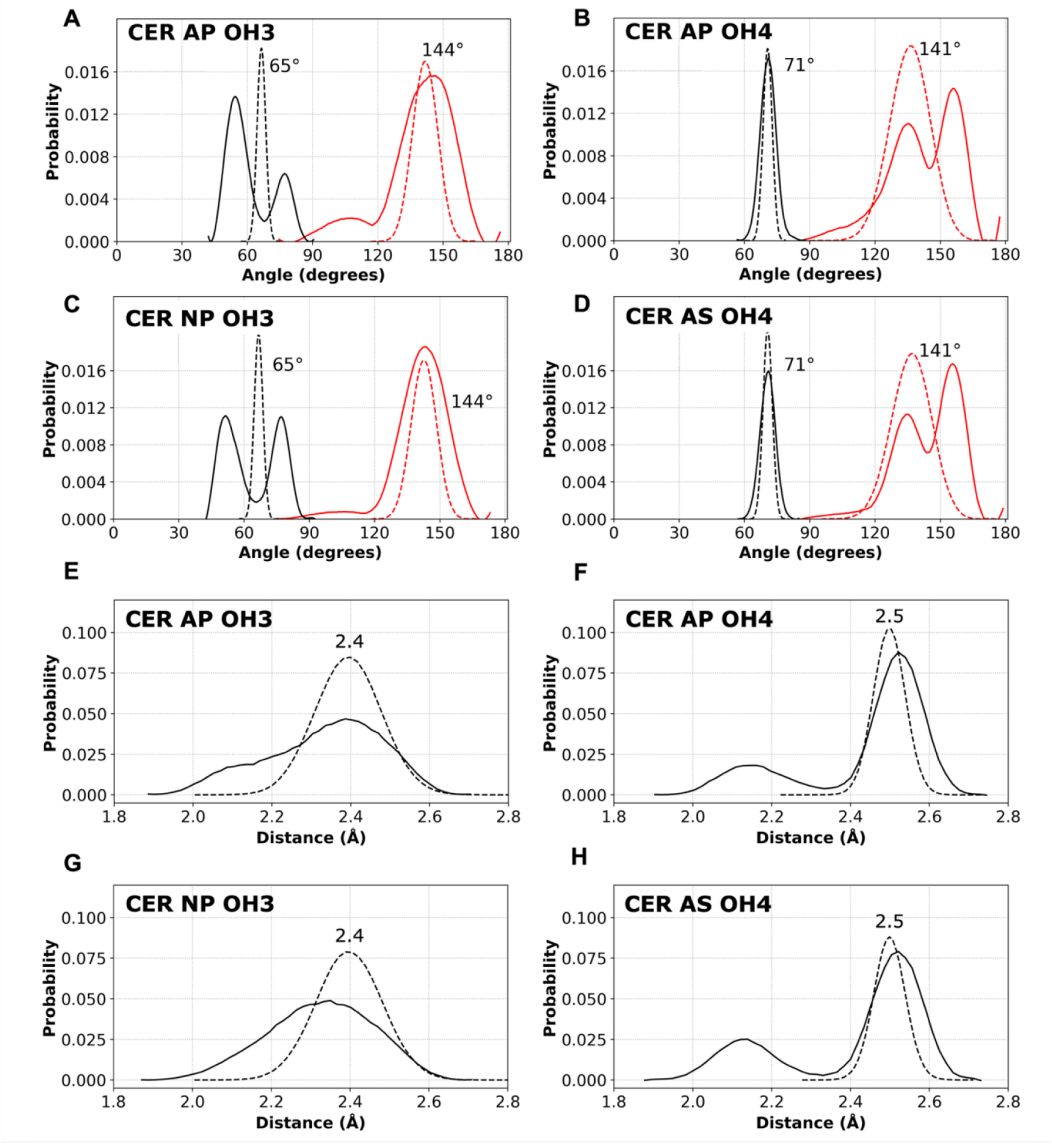
OH3 and OH4 angle (A–D) and bond length (E–H) distributions of pure CERs NP, AS, and AP bilayers from the preassembled AA simulations mapped to the CG level (solid lines) compared with self-assembled CG simulations (dashed lines), which used the OH1 nonbonded parameters for both OH3 and OH4. Angle distributions for TAIL–TAIL–OHn (*n* = 3 or 4) are in red with the CG peak centered at 144° for OH3 (A,C) and at 141° for OH4 (B,D). Angle distributions for MHEAD2–TAIL–OH3 (A,C) and AMIDE–TAIL–OH4 (B,D) are in black with the CG peaks centered at 65° and 71°, respectively. Bond length distributions are centered at 2.4 Å for TAIL–OH3 (E,G) and at 2.5 Å for TAIL–OH4 (F,H).

Bonded distributions from the AA simulations that have been mapped to the CG level are unimodal for the TAIL–OH3 bond length and two angles (TAIL–TAIL–OH3 and AMIDE–TAIL–OH4). Using the bonded coefficients (as well as the final nonbonded interaction parameters discussed later) in Table 3 to self-assemble a comparable pure bilayer produced a CG angle distribution that largely captured the AA behavior. However, the TAIL–OH4 bond length and angle distributions for MHEAD2–TAIL–OH3 and TAIL–TAIL–OH4 are bimodal for the AA bilayer. Here, the CG distribution falls between the two distinct AA peaks, still mostly capturing the general AA behavior.

Fitting bond angle distributions with multiple harmonic potentials could improve the match between AA and CG models but at the cost of reduced computational efficiency. However, based on previous studies,^29^ such a high level of agreement is often unnecessary for achieving acceptable consistency with structural properties from the CG simulations in comparison to the AA simulations. Thus, CG angle distributions representing bimodal AA distributions as a single peak are frequently used to maximize computational speed.^26^

### 3.3 Nonbonded Interaction Parameters

Nonbonded interaction parameters for OH3 and OH4 were assigned values from either OH1 or OH2, with the requirement that the same assignment of OH1 or OH2 parameters to OH3 and OH4 must be consistent across the CER NP, AS, and AP models. To assess the suitability of the OH1 or OH2 parameter choice, self-assembled CG bilayers of pure CER NP, AS, and AP, as well as mixtures of each CER in 1:0.5:1 molar ratios of CER:CHOL:FFA, were studied, and the results were compared with those from preassembled AA simulations of the same systems (Tables 1 and 2).

The structural metrics obtained from the bilayer simulations for both the pure and mixed bilayer systems showed minimal variation for any OH1 or OH2 assignments to OH3 and OH4, with the exception of pure CER AS, as shown in Tables 1 and 2. When OH2 parameters were assigned to OH4 in CER AS, the bilayer thickness exhibited a substantially higher standard deviation (16% of the mean) compared to less than 2% for all other OH1 and OH2 assignments (Table 1). This large variability was eliminated when the OH1 parameters were used for OH4.

While overall agreement between atomistic and CG models is good, some systematic differences in bilayer thickness are observed. For pure CER NP and CER AS bilayers, the CG models predict slightly thinner bilayers than the corresponding atomistic systems, whereas for CER AP the thicknesses are in close agreement (Table 1). Similarly for the ternary CER:CHOL:FFA systems reported in Table 2, the CG bilayers are consistently thinner than the comparable atomistic bilayers. This discrepancy may reflect differences in how the water–bilayer interface is represented, with the CG model’s larger, isotropic water beads penetrating more deeply into the headgroup region and broadening the interface, which in turn shifts the density profile peaks closer together.

In six-leaflet self-assembled multilayers of CER AP, CHOL, and FFA, the four inner leaflets—those not in contact with bulk water—were found to become disordered when OH2 parameters were assigned to the OH4 group but remained ordered when OH1 parameters were used (Figure 4). The ternary mixture of CER AS exhibited a similar behavior (data not shown). These differences in lamellar organization are evident in the structural metrics summarized in Table 4. Assigning OH2 parameters to OH4 in either CER AP or CER AS led to low order (*S*_2_ < 0.61), reduced central leaflet-pair thicknesses (<4.8 nm), elevated APL values (>0.41 nm^2^), and large tilt angles (≥24.6°), all consistent with disrupted lamellar packing. In contrast, using OH1 parameters produced highly ordered structures (*S*_2_ > 0.89), typical central leaflet-pair thicknesses (∼5.3 nm), normal APL values (∼0.34 nm^2^), and moderate tilt angles (10.4–12.2°). This pattern was also observed in six-leaflet multilayers of pure CERs AP and AS (Table 5): assigning OH2 to OH3 caused disorder, while OH1 preserved order. In fact, the disorder was so severe for pure CER AS with OH2 parameters assigned to OH4, and for pure CER AP with OH2 assigned to both OH3 and OH4, that stable structures could not be obtained; the simulation box width continued to fluctuate throughout the final 200 ns of the simulation.

**Figure 4:**
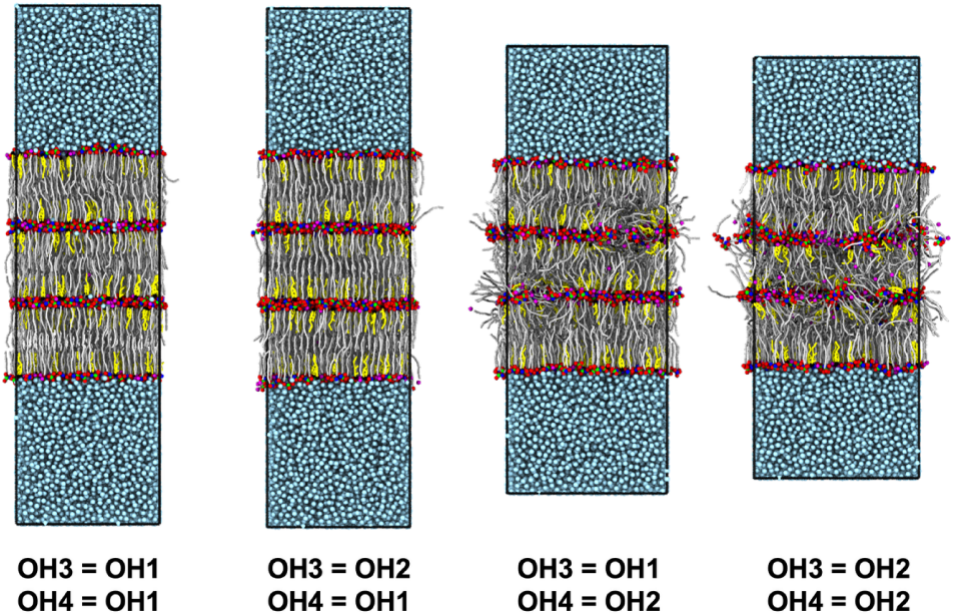
Snapshots of final configurations from self-assembled multilayer CG simulations of CER AP:CHOL:FFA mixtures with a 1:0.5:1 molar ratio with nonbonded interaction parameters from either OH1 or OH2 beads assigned to OH3 and OH4 beads (drawn to scale). Lipid backbones are represented as stick models (CER and FFA tails in gray and CHOL body in yellow) with headgroup beads rendered as spheres for CER AP (AMIDE in blue, MHEAD2 in green, and OH beads in red), FFA (HEAD in purple), and CHOL (CHEAD in black); water is light blue.

**Table 4:** Structural Parameters for Six-Leaflet Multilayers of CER:CHOL:FFA Mixtures with 1:0.5:1 Molar Ratio from Self-Assembled CG Simulations Using the Interaction Parameters for OH1 and OH2 Beads as Indicated to Parametrize the OH3 and OH4 CG Beads^a^

	OH1/OH2 assignment to						
CER	OH3	OH4	APL (nm^2^)	NLA (nm^2^)	Tilt Angle (°)	Leaflet-pair Thickness (nm)	S_2_
NP	OH1	–	0.337 ± 0.003	0.214 ± 0.002	10.8 ± 2.3	5.26 ± 0.05	0.92 ± 0.03
	OH2	–	0.340 ± 0.004	0.215 ± 0.003	11.2 ± 1.3	5.24 ± 0.04	0.91 ± 0.02
AS	–	OH1	0.335 ± 0.004	0.212 ± 0.002	12.2 ± 1.2	5.27 ± 0.08	0.89 ± 0.03
	–	OH2	0.415 ± 0.021	0.262 ± 0.013	24.6 ± 3.3	4.74 ± 0.34	0.61 ± 0.12
AP	OH1	OH1	0.338 ± 0.003	0.214 ± 0.002	10.6 ± 1.0	5.25 ± 0.04	0.92 ± 0.02
	OH1	OH2	0.457 ± 0.018	0.289 ± 0.011	26.5 ± 0.9	3.79 ± 0.27	0.44 ± 0.05
	OH2	OH1	0.347 ± 0.009	0.219 ± 0.006	10.4 ± 3.1	5.25 ± 0.05	0.91 ± 0.08
	OH2	OH2	0.466 ± 0.008	0.297 ± 0.006	29.9 ± 0.5	4.07 ± 0.08	0.39 ± 0.03
NS	–	–	0.339 ± 0.003	0.215 ± 0.002	11.7 ± 1.9	5.29 ± 0.04	0.90 ± 0.03

a APL and NLA are calculated for the inner four leaflets. Tilt angle, leaflet-pair thickness, and *S*_2_ are calculated for the central leaflet pair.

**Table 5:** Structural Parameters for Six-Leaflet Multilayers of Pure CER from Self-Assembled CG Simulations Using the Interaction Parameters for OH1 and OH2 Beads as Indicated to Parameterize the OH3 and OH4 CG Beads^a^

	OH1/OH2 assignment to						
CER	OH3	OH4	APL (nm^2^)	NLA (nm^2^)	Tilt Angle (°)	Leaflet-pair Thickness (nm)	S_2_
NP	OH1	–	0.440 ± 0.013	0.220 ± 0.006	12.9 ± 3.7	5.65 ± 0.15	0.95 ± 0.01
	OH2	–	0.430 ± 0.006	0.215 ± 0.003	11.1 ± 1.6	5.89 ± 0.22	0.93 ± 0.04
AS	–	OH1	0.465 ± 0.015	0.232 ± 0.008	20.5 ± 4.4	5.27 ± 0.24	0.95 ± 0.01
	–	OH2^b^					
AP	OH1	OH1	0.434 ± 0.003	0.217 ± 0.002	15.0 ± 1.6	5.79 ± 0.15	0.83 ± 0.03
	OH1	OH2	0.552 ± 0.008	0.276 ± 0.004	26.9 ± 0.6	4.72 ± 0.09	0.59 ± 0.02
	OH2	OH1	0.440 ± 0.006	0.220 ± 0.003	15.7 ± 2.9	5.81 ± 0.16	0.82 ± 0.05
	OH2	OH2^b^					
NS	–	–	0.453 ± 0.009	0.226 ± 0.004	17.8 ± 3.1	5.38 ± 0.21	0.95 ± 0.01

a APL and NLA are calculated for the inner four leaflets. Tilt angle, leaflet-pair thickness, and *S*_2_ are calculated for the central leaflet pair.

b An asterisk (*) designates systems that did not form stable structures as indicated by a constant simulation box width over the final 200 ns of simulation.

We note that while conformational distributions in these multilayers are expected to be similar across leaflets, a leaflet-by-leaflet analysis was not performed in this work. Related work by Shamaprasad et al.^39^ on multilayers of CER NS, CHOL, and FFA C24 in a 1:0.5:1 molar ratio showed that the numbers of extended acyl and sphingoid base chains were similar in the central leaflet pair, suggesting only minor leaflet-dependent variation.

In contrast to OH4, OH3 in CERs NP and AP is insensitive to whether OH1 or OH2 parameters were used in both the bilayer simulations (Tables 1 and 2) and multilayer simulations (Figure 4 and Tables 4 and 5). This insensitivity may not be surprising, given that OH1, like OH4, is positioned near the top of the headgroup, whereas OH2 lies deeper. From their positions near the top of the headgroup, OH1 and OH4 are more likely than OH2 to form hydrogen bonds with headgroups in opposing leaflets in multilayer systems or with bulk water in bilayers. The nonbonded interaction parameters for OH1 and OH2 reflect this difference in hydrogen bond directionality, making the OH1 parameters more appropriate for OH4. By contrast, such directionality differences may be less relevant for OH3, which is located below OH2 on the sphingoid base chain.

Based on these results, OH1 nonbonded interaction parameters were selected for both OH3 and OH4. This assignment yields CG models for CERs NP, AS, and AP that reproduce AA bilayer simulations and align with experimental observations of well-ordered SPP in their mixtures with CHOL and FFA, without requiring new nonbonded groups to be parametrized.

An important additional finding is that bilayer simulations are markedly less sensitive to differences in the nonbonded interaction parameters than simulations involving multiple leaflets with minimal bulk water contact. This likely reflects the stabilizing influence of hydrogen bonding between lipid headgroups and water in bilayers, which can compensate for less optimal lipid–lipid interactions. In the inner leaflets of multilayers, where such water-mediated stabilization is mostly absent because water content is low (<0.35 water molecules/lipid), the effects of lipid–lipid interactions, which are directly related to the CG interaction parameters, become much more pronounced.

### 3.4 Effect of CER Subclass on Lamellar Organization and Structure

Using the newly developed CG models for CERs NP, AP, and AS, we examined how the CER subclass affects lipid organization and structure. Table 6 compares structural properties across CER subclass, including leaflet-pair thickness, *S*_2_, tilt angle, and interdigitation of the central leaflet pair as well as APL, NLA, and the percentage of CERs in extended conformations in the inner four leaflets.

**Table 6:** Structural Parameters for Self-Assembled Six-Leaflet Multilayers of Pure CER and CER:CHOL:FFA Mixtures with a 1:0.5:1 Molar Ratio^a^

System	CER	APL (nm^2^)	NLA (nm^2^)	Tilt Angle (°)	Leaflet-pair Thickness (nm)	S_2_	Interdigitation (nm)	CER in extended conformation (%)	Water molecules per lipid
Pure	NS	0.453 ± 0.009	0.226 ± 0.004	17.8 ± 3.1	5.38 ± 0.21	0.95 ± 0.01	0.87 ± 0.03	29.5 ± 2.9	0.13 ± 0.03
	NP	0.440 ± 0.013	0.220 ± 0.006	12.9 ± 3.7	5.65 ± 0.15	0.95 ± 0.01	0.94 ± 0.03	28.8 ± 0.6	0.26 ± 0.02
	AS	0.465 ± 0.015	0.232 ± 0.008	20.5 ± 4.4	5.27 ± 0.24	0.95 ± 0.01	0.82 ± 0.02	24.7 ± 0.6	0.19 ± 0.04
	AP	0.434 ± 0.003	0.217 ± 0.002	15.0 ± 1.6	5.79 ± 0.15	0.83 ± 0.03	1.25 ± 0.23	30.0 ± 0.8	0.23 ± 0.05
Mixture	NS	0.339 ± 0.003	0.215 ± 0.002	11.7 ± 1.9	5.29 ± 0.04	0.90 ± 0.03	1.06 ± 0.03	33.1 ± 2.8	0.36 ± 0.02
	NP	0.337 ± 0.003	0.214 ± 0.002	10.8 ± 2.3	5.26 ± 0.05	0.92 ± 0.03	1.14 ± 0.01	35.8 ± 0.9	0.34 ± 0.02
	AS	0.335 ± 0.004	0.212 ± 0.002	12.2 ± 1.2	5.27 ± 0.08	0.89 ± 0.03	0.97 ± 0.03	32.9 ± 2.5	0.34 ± 0.04
	AP	0.338 ± 0.003	0.214 ± 0.002	10.6 ± 1.0	5.25 ± 0.04	0.92 ± 0.02	1.13 ± 0.01	37.1 ± 1.0	0.28 ± 0.03

a APL, NPL, and the percentage of CERs in the extended conformation are for the four inner leaflets. Tilt angle, leaflet-pair thickness, *S*_2_, and interdigitation are for the central leaflet pair.

In the pure CER multilayers, APL values are consistent across CERs (0.43–0.47 nm^2^). CER AP exhibits significantly lower chain order (*S*_2_ = 0.83) than the other CERs (*S*_2_ = 0.95), consistent with experimental observations of less ordered lamellar packing—hexagonal rather than the orthorhombic packing seen for CERs NS and AS—and its larger headgroup (containing 4 rather than 2 or 3 OH groups) with strong hydrogen bonding.^67^ A general trend is observed in which the phytosphingosine CERs have larger leaflet-pair thicknesses, smaller tilt angles, and larger interdigitation than do their sphingosine counterparts, although not all differences were statistically significant. CER AS exhibited the lowest percentage of CERs in the extended conformation (24.7%), compared with ∼30% for the others.

For multilayers of CER mixed with CHOL and FFA, the structural metrics (APL, NLA, tilt angle, leaflet-pair thickness, and *S*_2_) are generally similar, except for CER AS, which had reduced interdigitation (0.97 nm vs ∼1.13 nm for the others). This may be related to its slightly larger, though not statistically significant, tilt angle (12.2° vs ∼10.7°). CER AP exhibited the highest percentage of CERs in the extended conformation (37.1%), and the phytosphingosine CERs generally adopt the extended conformation more often than their sphingosine counterparts, although the difference is not statistically significant for CER NP relative to CER NS.

Leaflet-pair thicknesses in the multilayer CER mixtures (5.24–5.27 nm) closely match the repeat distances observed experimentally for CERs NS, AS, and AP (5.27–5.40 nm) in equimolar mixtures with CHOL and either a five-component FFA mixture or only FFA C24.^11,15,68^ Since equimolar mixtures were studied, all of these mixtures also contained a separate crystalline CHOL phase.^11,15,68^ Experimental SPP-like repeat distances for CER NP with CHOL and FFA C24 (5.24 to 5.56 nm) are also similar to the leaflet-pair thickness of the simulations.^10,13,68^ However, other lamellar phases (e.g., 4.26 and 3.72 nm) are present in the CER NP experiments (as well as crystalline CHOL), making the composition of the SPP-like phase uncertain (and perhaps different from those in the simulations).

Although the APL of the CER mixtures with CHOL and FFA is smaller than that of the corresponding pure CER systems, the NLA, which accounts for the size differences of CER, CHOL, and FFA, is nearly identical for all of the multilayer systems. With the exception of CER AP, the mixtures exhibit lower chain ordering (*S*_2_ ∼ 0.90) than their pure counterparts (*S*_2_ = 0.95), along with reduced leaflet-pair thickness. Finally, the percentage of CERs in the extended conformation is higher in the mixtures than in the corresponding pure systems: an increase of ∼3% for CER NS (which has only OH1 and OH2 groups) and ∼7–8% for CER NP, AS, and AP (which contain one or both of the OH3 and OH4 groups).

Overall, the results in Table 6 highlight how differences in CER headgroups affect the structure of the central leaflet pair of pure CERs, and how the addition of CHOL and FFA modulates these effects. Pure CER systems show distinct structural characteristics, with CER AP favoring extended conformation and interdigitation and CER AS showing greater tilt and compactness. Adding CHOL and FFA promotes more compact, upright, and interdigitated structures and increases the proportion of CERs in extended conformations, all of which are largely insensitive to the CER headgroup.

## 4 Conclusions

This work extends the MS-IBI CG model of CER NS to three additional CER subclasses that are abundant in human SC: CERs NP, AP, and AS. By leveraging their structural similarities to CER NS and using nonbonded interaction parameters for OH beads in CER NS to describe those in the other CERs, new CG models were developed with minimal additional parametrization, further demonstrating the transferability of MS-IBI-derived models.

Self-assembled CG bilayers of pure CERs and their ternary mixtures with CHOL and FFA were studied, and the resulting structures were validated against atomistically simulated bilayers, confirming that the new CG models reproduce key structural features for each CER subclass. With the CG models developed, six-leaflet CG multilayers of both pure CER and ternary mixtures with CHOL and FFA were then studied, enabling investigation into how the different CER headgroups affect the lamellar structure in the absence of the water interface. In the pure CER multilayers, headgroup-dependent differences were observed in leaflet-pair thickness, lipid packing, molecular tilt, and CER conformation for the inner leaflets that are not exposed to bulk water. These differences were largely eliminated when the CERs were mixed with CHOL and FFA, consistent with the experimental observations.

Through this work, we have enlarged the catalog of MS-IBI CG lipid models using a streamlined approach for generating new CG models. The resulting models expand the toolkit for simulating skin lipid systems that more accurately represent human SC. Simulations utilizing these models can complement experimental studies by providing molecular-scale insights that are difficult or impossible to obtain experimentally.

## Supplementary Material



## Notes

The statistical procedures used to calculate standard deviations for reported properties; a table with the complete coarse-grained bonded interaction parameters (bond lengths, bond force constants, bond angles, and angle force constants) (PDF)

## #

Memorial Sloan Kettering Cancer Center, New York, NY 10021, USA
