# Supplementary material for: New Coarse-Grained Models for Stratum Corneum Ceramides Reveal Headgroup-Dependent Structural Organization
Source: J Phys Chem B. 2025 Nov 13;129(47):12167–78. doi: 10.1021/acs.jpcb.5c05845 (PMC12670428; doi:10.1021/acs.jpcb.5c05845)
Supplement: Supplementary file 1 [file jp5c05845_si_001.pdf]

Supporting Information

New Coarse-Grained Models for Stratum Corneum Ceramides Reveal Headgroup-Dependent Structural Organization

Chloe O. Frame[1], Parashara Shamaprasad[1], Shubham Deshpande[2], Co D. Quach[1], Lingfeng Gui[2], Christopher R. Iacovella[1,#], Annette L. Bunge[3], Clare McCabe[1,2*]

[1] Department of Chemical and Biomolecular Engineering, Vanderbilt University, Nashville, TN 37235, USA
[2] School of Engineering and Physical Science, Heriot-Watt University, Edinburgh, UK
[3] Department of Chemical and Biological Engineering, Colorado School of Mines, Golden, CO 80401, USA

*Corresponding author

Contact information for corresponding author:
Dr. Clare McCabe, Bicentennial Professor
EH14 4AS
Heriot-Watt University
Edinburgh, Scotland, UK
Email: c.mccabe@hw.ac.uk

[#] Current address: Memorial Sloan Kettering Cancer Center, New York, NY, 10021, USA
[*]Corresponding author email: c.mccabe@hw.ac.uk

Standard deviation calculations

Except for tilt angle, results are reported as the mean and standard deviation (s) of the mean values of the frames from each of three or four trials (details listed in the Methods in the main text) with different randomized initial configurations as follows:

$$s = \sqrt{\frac{1}{I}\sum_{i=1}^{I} s_i^2} \tag{S1.1}$$

where s_i is the standard deviation of the mean values from all frames in trial i and I is the number of different trials. Because tilt angle is a per-lipid quantity, reported standard deviations are the standard deviations of the lipids in each leaflet pooled across all leaflets in all frames of the four trials calculated as

$$s = \sqrt{\frac{1}{IJK}\sum_{i=1}^{I}\sum_{j=1}^{J}\sum_{k=1}^{K} s_{ijk}^2} \tag{S1.2}$$

where s_{ijk} is the standard deviation of the tilt angles in leaflet k of frame j in trial i, I is the number of trials, J is the number of frames in each of the trials, and K is the number of leaflets in each frame.

Table S1: Bonded interaction parameters for the CER: r_0 is the length and k_r is the harmonic force constant for the bonds between adjacent CG beads; θ_0 is the angle and k_0 is the harmonic force constant for the bond angle between three connected beads.

**Bond**	r_0 **[Å]**	k_r **[kcal/(mol Å^2)]**
TER2-TAIL	3.41	35.6
TAIL-TAIL	3.92	20.9
TAIL-AMIDE	3.01	103.9
TAIL-MHEAD2	3.62	27.6
TAIL-OH3	2.40	93.0
TAIL-OH4	2.50	542.0
AMIDE-MHEAD2	2.92	190.0
MHEAD2-OH1	2.33	45.3
MHEAD2-OH2	2.07	152.0
**Angle**	θ_0 **[degrees]**	k_0 **[kcal/(mol-degrees^2)×10^3]**
TER2-TAIL-TAIL	155.0	2.41
TAIL-TAIL-TAIL	158.0	1.92
TAIL-TAIL-AMIDE	158.0	1.92
TAIL-TAIL-MHEAD2	158.0	1.92
TAIL-TAIL-OH3	143.8	16.5
TAIL-TAIL-OH4	141.0	4.88
MHEAD2-AMIDE-TAIL	106.0	8.84
AMIDE-MHEAD2-TAIL	107.0	1.62
AMIDE-MHEAD2-OH1	109.0	2.53
AMIDE-MHEAD2-OH2	105.0	3.29
AMIDE-TAIL-OH4	71.0	149
MHEAD2-TAIL-OH3-a[a]	52.2	70.4
MHEAD2-TAIL-OH3-b[a]	77.3	97.5
TAIL-MHEAD2-OH1	128.0	1.74
TAIL-MHEAD2-OH2	69.0	110
OH1-MHEAD2-OH2	115.0	3.72

[a] The MHEAD2-TAIL-OH3 angles are characterized by two distributions, labeled as a and b.
